# The mitochondrial genome of parasitic wasp: *Anisopteromalus calandrae* (Howard, 1881) (Hymenoptera: Pteromalidae)

**DOI:** 10.1080/23802359.2021.1942257

**Published:** 2021-06-21

**Authors:** Zhengyu Huang, Haiyan Dai, Qinlu Lin, Bin Zhang

**Affiliations:** a National Engineering Laboratory for Rice and By-product Deep Processing, College of Food Science and Engineering, Central South University of Forestry and Technology, Changsha, China; b Department of Anatomy and Neurobiology, Biology Postdoctoral Workstation, School of Basic Medical Sciences, Central South University, Changsha, China; c State Key Laboratory of Genetic Resources and Evolution, Kunming Institute of Zoology, Chinese Academy of Sciences, Kunming, China; d Shanghai Clinical Research Center Co., Ltd., Shanghai, China; e Shanghai Engineering Research Center of Biobank, Shanghai, China

**Keywords:** *Anisopteromalus calandrae*, Hymenoptera, mitochondrial genome, parasitic wasp

## Abstract

The parasitic wasp *Anisopteromalus calandrae* is a natural enemy of numbers store product pests. The mitochondrial genome (mitogenome) of *A. calandrae* was obtained by second-generation sequencing. The assembled mitogenome of *A. calandrae* is 15,954 bp long (GenBank accession: MW817149) and contains 37 typical animal mitochondrial genes. The order of the mitochondrial genes is identical to that of another species of Chalcidoidae (*Pteromalus puparum*). All protein-coding genes start with ATN codons, and end with TAA, except NAD4 and NAD5 with T.

The *Anisopteromalus calandrae* (Howard) is a well-known natural enemy of numbers important coleopterous stored product pests (Schöller et al. 2006), by parasitizing their larval or pupal stages. The cosmopolitan parasitic wasp has been investigated in biological control (Schöller et al. 2006), life-history traits (Narongplian et al. 2011), and other topics (Bressac et al., 2009). Data obtained from molecular fragments deepen the understanding of classification (Baur et al. 2014). This mitochondrial genome would supply more information on understanding its special character.

Samples of *A*. *calandrae* were originally collected alive from the culture flask of *Sarocladium oryzae* in National Engineering Laboratory for Rice and By-product Deep Processing, Central South University of Forestry and Technology, Changsha, Hunan province, China (28.14° N, 112.20° E). Samples are deposited at the Grain Insect specimens Museum (NEL6-19), Central South University of Forestry and Technology, China.

Whole genomic DNA was extracted with the CTAB method. The qualified DNA was sequenced using Illumina Novaseq. The clean reads were assembled using SPAdes (Version 3.13.1) (Bankevich et al. 2012). MITOS was performed to annotate the gene structure with five Invertebrates and other default parameters (Bernt et al. 2013).

The mitochondrial genome of *A. calandrae* consists of 15,954 bp in length with 82.9% of A + T (accession number MW817149), containing 13 PCGs (ATP6, ATP8, COXI-III, NAD1-6, NAD4L, and CYTB), two rRNAs genes (srRNA and lrRNA), and interspersed 22 tRNAs genes. The order of the mitochondrial genes is identical to that of another species of Chalcidoidae (*Pteromalus puparum*), including “cox1-trnL2-cox2-trnK-trnD-atp8-atp6-cox3” and “trnA-rrnL-trnL1-nad1” block shared in chalcidoid (Shimada and Fujii 1985; Yan et al. 2019). Furthermore, a non-coding region was found inserted between “trnA-rrnL” and “trnA2-CYTB.”

All of the 13 PCGs start with a typical ATN codon: six with ATT (NAD2, NAD3, ATP8, NAD5, NAD4l, and AND6), five with ATG (COX3, ATP6, COX1, NAD4, and COB), and two with ATA (COX2 and NAD1). Eleven PCGs stop with the complete termination codons TAA. The two exceptions are NAD4 and NAD5, which use T as a stop codon.

The second structure of 20 tRNA was predicted as typical cloverleaf secondary structures, except TrnS1 (AGA), which lacked a D ring and dihydrouridine (DHU) arm.

The phylogenetic relationships were reconstructed using Maximum Likelihood (ML) with MEGA7 (Kumar et al. 2016) and Bayesian inference (BI) with BEAST V 1.8.2 (Drummond et al. 2012) based on 13 PCGs dataset with nine Chalcidoidea from GenBank. The ML analyses were carried with the NJ model and 1000 bootstrap replications. Each BEAST analysis performed a lognormal relaxed clock model with 100 million generations (Gernhard 2008). The ML and BI analyses resulted in very similar topologies, and BI is better supported the relationships (Figure 1). Both BI and ML tree strongly supported the sister relationship between *A. calandrae* and *P. puparum* (PP = 1.00, BS = 100), then sister to *Tamarixia radiata* (PP = 1.00, BS = 0.64). The clade of the three wasps is a well-supported sister to a clade comprising of *Aenasius arizonensis* and *Encyrtus infelix*, *Trichogramma dendrolimi*, and *Megaphragma amalphitanum* in BEAST tree (PP = 0.94, Figure 1). The other nodes were weakly supported, and more taxon sequences would be necessary.

**Figure 1.**
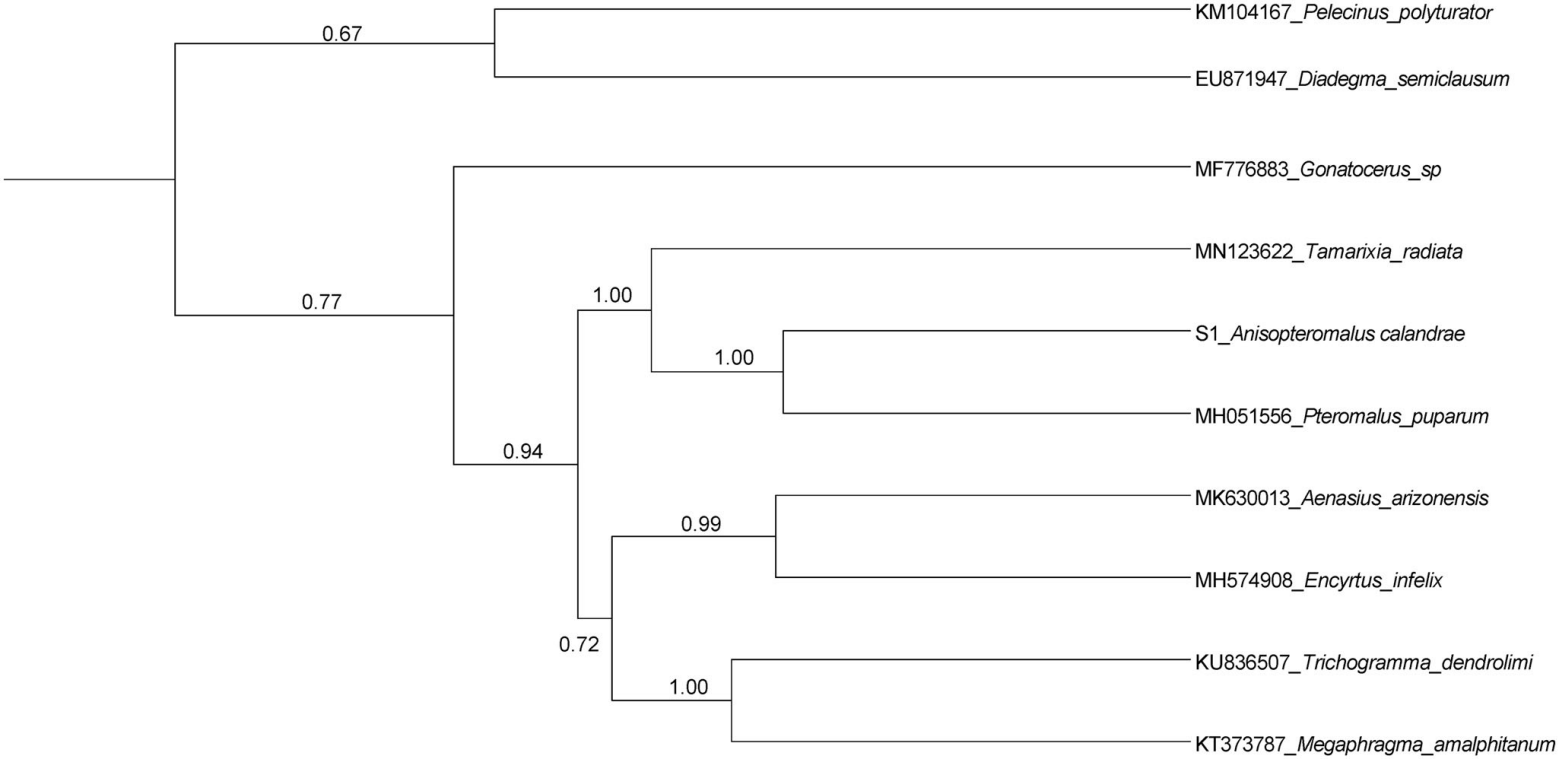
BEAST tree of *Anisopteromalus calandrae* and other nine Chalcidoidea species based on 13 PCGs data. Node numbers indicate posterior probabilities of the BEAST analyses. Genbank accession number for sequences download from GenBank were listed ahead each species name.

## Data Availability

The data that support the findings of this study are openly available in “NCBI” at https://www.ncbi.nlm.nih.gov/, reference accession number MW817149.
